# Structural Variants: Mechanisms, Mapping, and Interpretation in Human Genetics

**DOI:** 10.3390/genes16080905

**Published:** 2025-07-29

**Authors:** Shruti Pande, Moez Dawood, Christopher M. Grochowski

**Affiliations:** 1 Department of Molecular and Human Genetics, Baylor College of Medicine, Houston, TX 77030, USA; shruti.pande@bcm.edu (S.P.); moez.dawood@bcm.edu (M.D.); 2 Human Genome Sequencing Center, Baylor College of Medicine, Houston, TX 77030, USA; 3 Medical Scientist Training Program, Baylor College of Medicine, Houston, TX 77030, USA

**Keywords:** structural variations (SVs), SV mutagenesis mechanisms, next-generation sequencing, multi-omics, SV callers

## Abstract

Structural variations (SVs) represent genomic variations that involve breakage and rejoining of DNA segments. SVs can alter normal gene dosage, lead to rearrangements of genes and regulatory elements within a topologically associated domain, and potentially contribute to physical traits, genomic disorders, or complex traits. Recent advances in sequencing technologies and bioinformatics have greatly improved SV detection and interpretation at unprecedented resolution and scale. Despite these advances, the functional impact of SVs, the underlying SV mechanism(s) contributing to complex traits, and the technical challenges associated with SV detection and annotation remain active areas of research. This review aims to provide an overview of structural variations, their mutagenesis mechanisms, and their detection in the genomics era, focusing on the biological significance, methodologies, and future directions in the field.

## 1. Introduction to Structural Variations

Structural variations (SVs) are an important class of human genomic variations. SVs are genomic rearrangements ranging in size from 50 base pairs to several million base pairs [1]. These SVs can create novel breakpoint junctions that have been associated with normal genomic variation [2], Mendelian diseases [3,4], or complex disease traits [3,5] and contribute significantly to genomic diversity. SVs can be simple genomic rearrangements in the form of copy-number variations (CNVs) like deletions or duplications [4] or complex genomic rearrangements (CGRs) based upon the number of breakpoint junctions, with the latter having more than two breakpoint junctions (ex. duplication–normal–duplication, duplication–triplication/inversion–duplication, etc.) [5,6,7,8,9].

SVs/CNVs are as important as single-nucleotide variations (SNVs) in assessing the differences between individuals and considered a major driving force behind the rapid evolution that occurred over time and continues to occur amongst different species lineages [10]. These genomic alterations include copy-neutral events (balanced inversions, translocations, copy-neutral intra- and inter-chromosomal insertions; and copy-neutral Loss of Heterozygosity (cnLOH) [9] or CNVs including deletions, duplications, triplications, or other higher-order amplifications [11] that have an impact on gene dosage levels [12].

Based upon the localization of breakpoints, SVs can be classified into either recurrent or non-recurrent rearrangements. Recurrent SVs arising through non-allelic homologous recombination (NAHR) are characterized by consistent size, breakpoint architecture, and genomic content across unrelated individuals [3,11,12]. This rearrangement mechanism can be driven by low-copy repeats (LCRs), segmental duplications (SDs), or repetitive sequences (*Alu* elements) serving as homologous recombination substrates, leading to recurrent events with clustered breakpoints [11]. LCRs are defined as intra-chromosomal duplications ≥10 kb in length and with ≥97% sequence similarity [13]. LCR genome-wide distribution patterns show overlap with regions that frequently undergo genomic rearrangements, causing recurrent deletions/duplications associated with genomic disorders [12]. One set of widely studied examples of NAHR-mediated genomic rearrangements includes the genomic disorders at the 17p11.2 locus. NAHR between the direct and inverted LCRs flanking the dosage-sensitive *RAI1* gene contributes to a common recurrent rearrangement of ~3.4 Mb, leading either to a heterozygous duplication causing Potocki–Lupski syndrome (PTLS; MIM: 610883) [14] or a deletion leading to Smith–Magenis syndrome (SMS; MIM: 182290) [15].

In contrast to common recurrent genomic rearrangements, non-recurrent events [3] vary in size but encompass the dosage-sensitive gene either entirely or partially, but individuals with similar clinical phenotypes often share a minimal region of overlap within these variants. Non-homologous end joining (NHEJ) is one of the most common repair mechanisms implicated for double-strand breaks (DSBs), in which the broken DNA ends are ligated together by enzymes [16,17]. Microhomology-mediated end joining (MMEJ) is an alternative end-joining pathway that uses short micro-homologous sequences (5–25 bp) at the breakpoints for alignment [18,19]. Finally, replicative repair/recombination processes that occur during DNA replication and often involve replication stress or fork collapse, such as break-induced replication (BIR), fork stalling and template switching (FoSTeS) [20], and microhomology-mediated break-induced replication (MMBIR), are amongst the other SV mutagenesis mechanisms [18].

SVs can also be categorized into other catastrophic genomic events forming combinations of multiple complex SV types including chromoanagenesis [8,19,21], chromothripsis [22,23], and chromoplexy [24,25]. Although the biological outcomes of these three complex chromosomal events are similar, their underlying molecular mechanisms are distinct [21]. Chromothripsis is the localized shattering of a chromosome, followed by the random reassembly of its fragments, leading to complex genomic rearrangements, and has been identified in cancers [26], developmental disorders [27], and even in apparently asymptomatic individuals [28]. Breakpoint junction analysis of shattered chromosomal fragments from chromothripsis shows classical NHEJ or an alternative form of end joining (alt-NHEJ) as the mechanisms of reassembly [29]. Chromoplexy is characterized by the interconnected occurrence of multiple inter- and intra-chromosomal translocations and deletions, arising from DSBs, and can involve multiple chromosomes, resulting in derivative chromosomes with minimal or no copy-number alterations [25]. Chromoanasynthesis is a replication-based complex rearrangement process characterized by copy-number gains (duplications and triplications) in combination with deletions and copy-neutral chromosomal segments [30]. Breakpoint junction analysis shows micro-homology and template insertions, suggestive of defective DNA replicative mechanisms and error-prone DNA replication pathways such as FoSTeS and MMBIR [18].

SVs represent a major component of human genomic variation, influencing both genomic diversity and disease susceptibility [1,31]. To aid in distinguishing pathogenic SVs from polymorphisms, several population-level databases have been developed that catalog SVs observed in healthy individuals, including Database of Genomic Variants (DGV), which has a comprehensive collection of SVs observed in control populations [31]; gnomAD-SV, providing population-level allele frequencies for SVs across diverse ancestries [1]; dbVAR, an NCBI-hosted database curating large-scale genomic variation [32]; Human Genome Structural Variation Consortium (HGSVC) [33]; and SVAFotate [34]. These databases continue to improve the resolution and annotation of SVs across populations.

In this review, we aim to provide a comprehensive overview of SVs, emphasizing their clinical relevance, challenges in interpretation, and the evolution of detection methodologies over time. We begin by outlining the concepts and clinical implications of SVs, followed by a detailed view of the various approaches used to identify SVs, ranging from early cytogenetic techniques to current state-of-the-art genomic technologies. Finally, we discuss emerging trends, future directions, and the potential approaches to enhance SV discovery and interpretation in both research and clinical settings.

## 2. Clinical Relevance

**Figure 1:**
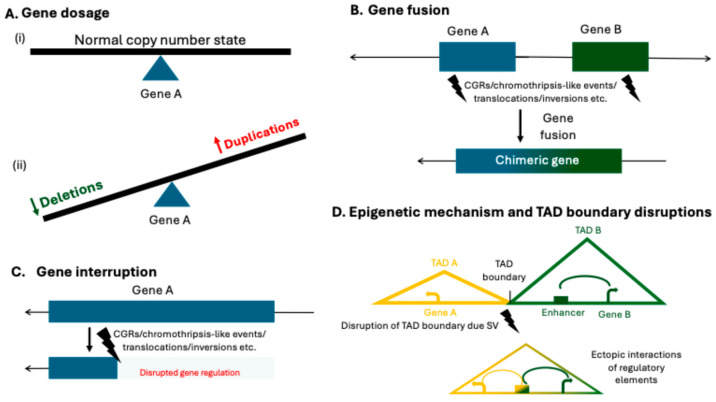
Consequences of SVs on gene structure. (**A**) Schematic representation of normal gene dosage for gene A, illustrated with a black line. When there is neither a deletion or duplication of a gene (the state is normal), the dosage of that gene is steady (i). Gene dosage alterations due to copy-number variations are illustrated by the see-saw dips: a decrease in dosage is shown in green for deletions, and an increase is shown in red for duplications, showing the direction of gene dosage change when this type of SV occurs (ii). (**B**) Illustration of gene fusion events resulting from complex genomic rearrangements or chromothripsis-like phenomena, leading to the formation of a novel chimeric gene by joining two previously separate genes, A (blue) and B (green). (**C**) Depiction of gene disruption caused by SVs such as deletions, duplications, insertions, inversions, or complex genomic rearrangements which physically interrupt the coding sequence or regulatory regions of gene A; the lighter bar on the bottom half of figure shows the disruption of gene regulation. (**D**) SVs altering the 3D genomic architecture by repositioning regulatory elements such as enhancers, silencers, or insulators relative to genes A (yellow) and B (green), potentially leading to ectopic regulatory interactions (combination of yellow and green).

Genomic disorders often arise from structural rearrangements driven by the genome’s unique architectural features (LCRs, SDs, *Alu* elements) which predispose these regions to genomic instability. These rearrangements can involve dosage-sensitive genes, resulting in either loss or gain of gene dosage, ultimately altering gene expression and contributing to disease [3,12,35] (Figure 1A).

SVs like translocations, inversions, interstitial deletions, or CGRs can lead to a gene fusion event by joining two originally separate genes by forming a novel chimeric gene (Figure 1B). The fusion can lead to a hybrid protein or alter the regulatory control of one or both genes and a novel disease mechanism, frequently identified in cancers [36], including the *ETV6-NTRK3* fusion gene associated with secretory breast cancer [37], *BCR-ABL1* in chronic myeloid leukemia [38], and chromothripsis-associated multiple gene fusions [26]. Gene interruption is caused by the physical disruption of a gene’s coding sequence or regulatory elements due to SVs such as deletions, duplications, insertions, inversions, or CGRs (Figure 1C). Such disruption can lead to loss of function, haploinsufficiency, dominant-negative effects, or altered expression patterns, thereby contributing to genetic disease or cancer [4,39]. SVs disrupting inter- and intra-genic regions or regulatory regions have been frequently found to be associated with several developmental disorders as well as cancers [40,41].

The role of SVs contributing to disease, partly by disrupting the three-dimensional organization of the genome, the spatial chromatin architecture, is an active area of research [42,43]. SVs may also interfere with topologically associating domains (TADs), the key elements of the dynamic regulatory architecture [43,44]. SVs can disrupt this spatial organization of the genome, repositioning key regulatory elements such as enhancers, silencers, and insulators, or create ectopic interactions between genes and regulatory elements that are normally insulated, thereby interfering with their normal interactions with target genes (Figure 1D). This mis-regulation can result in aberrant gene expression and has been studied with X-linked acrogigantism (X-LAG; MIM: 300942), which involves disruption of the *GPR101* locus [45]; congenital limb malformation associated with tandem duplications at the *LBX1/FGF8* locus [46]; human limb malformations caused by SVs altering the structure of the TAD-spanning *WNT6/IHH/EPHA4/PAX3* locus [43]; and disruption of the *Epb41l4a* TAD boundary associated with neurological and neurodevelopmental phenotypes [47].

## 3. Interpretation of Structural Variants

Understanding the clinical relevance of CNVs/SVs is complex (particularly those encompassing non-coding, inter-genic, or complex genomic rearrangements) and is continually evolving [48]. The vast majority of CNVs or SVs identified in clinical, or research settings are unique to an individual, and they often lack clear or consistent associations with specific clinical phenotypes, thereby making the interpretations challenging [48]. Accurate clinical interpretation of SVs requires a systematic and standardized approach involving evaluation of the genomic content (dosage-sensitive genes, regulatory regions, or highly conserved regions) and extent. It also involves cross-referencing information from genomic databases including DECIPHER [49] and ClinVar [50] and population databases like gnomAD-SV and DGV, as well as published case studies and guidelines from professional organizations like American College of Medical Genetics and Genomics (ACMG) and the Clinical Genome Resource (ClinGen) [48]. ClinGen, ClinVar, and DECIPHER host extensive repositories of known and recurrent structural variants (SVs) associated with well-characterized microdeletion and microduplication syndromes, and they are continuously updated to include SVs linked to single-gene disorders as well, thereby providing a valuable resource for analysts.

## 4. Methods for Structural Variant Detection

**Figure 2:**
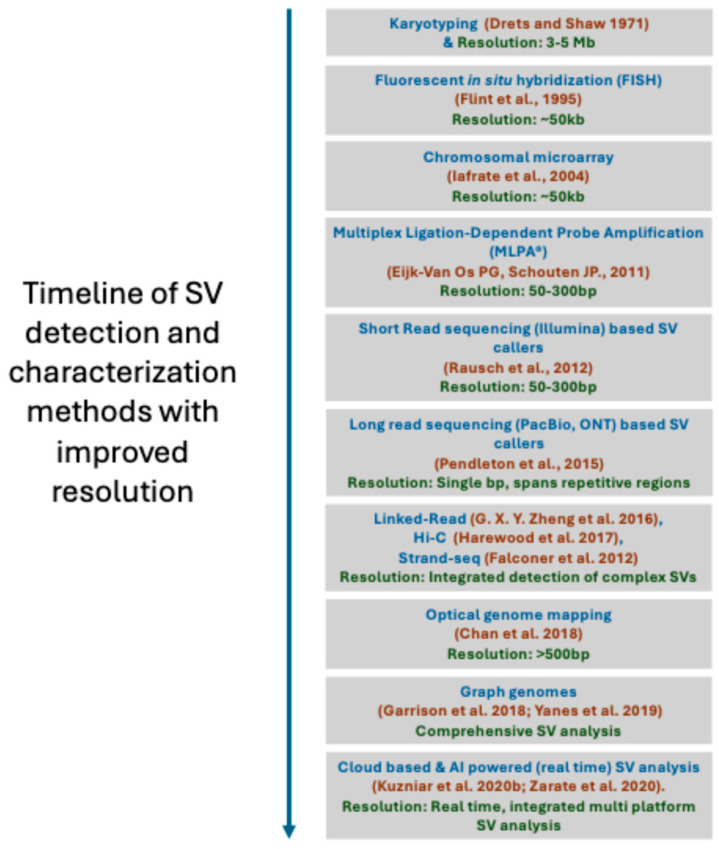
Timeline of SV detection methods. Timeline illustrating the evolution of major SV detection methods, annotated with the year each method was first introduced. The progression highlights improvements in resolution over time, enabling more accurate identification, interpretation, and characterization of both simple and complex genomic rearrangements.

The field of cytogenetics has witnessed significant progress since the discovery of human diploid chromosomes in 1956 [51]. These advancements have occurred not only in technology but also in our understanding of structural variation mutagenesis mechanisms, gene dosage, genomic disorders, and genotype–phenotype correlations [12,52]. The timeline of emerging SV analysis methods is shown in Figure 2.

### 4.1. Karyotyping

G-banded karyotyping, a conventional cytogenetic method using trypsin digestion and Giemsa staining, can detect aneuploidies, polyploidies, mosaicism, and structural variations, contributing to genomic diagnoses in up to 3% of cases [53]. The major disadvantages of conventional karyotyping include relatively low resolution (up to 5–10 Mb), a long turnaround time, and the need for dividing cells [54,55]. This method is still usedfor detection of certain chromosomal abnormalities such as marker chromosomes [56].

### 4.2. Chromosomal Microarray

High-resolution chromosomal microarray (CMA) has emerged as a gold standard and first-tier test for individuals with intellectual disability, autism spectrum disorder, and multiple congenital anomalies, increasing the yield of cytogenetic testing up to 19% [57,58]. CMA can identify CNVs as small as 20–50 kb and enable detection of copy-number variations affecting single genes [59]. Microarray testing can uncover novel, or candidate genes associated with a clinical condition through gene dosage analysis, thereby proving the test’s diagnostic capability for disease gene discovery [58,60]. The combination of karyotyping and CMA has demonstrated significant effectiveness in SV discovery for prenatal testing [61].

### 4.3. Targeted CNV Detection

Fluorescence in situ hybridization (FISH) [62] and multiplex ligation-dependent probe amplification (MLPA) [63] can identify smaller chromosomal copy-number variations. However, these techniques are targeted testing modalities, in contrast to genome-wide CNV detection, and are limited to cases with a specific clinical suspicion or the analysis of sub-telomeric regions, which are commonly affected in children with developmental impairments [62,64].

### 4.4. Optical Genome Mapping

Optical genome mapping (OGM) is a technique capable of detecting SVs that are challenging to identify using short-read sequencing or other conventional methodologies. It works by fluorescently labeling ultra-long, high-molecular-weight linearized DNA molecules at specific sites, generating a high-resolution map of genomic variations, including repetitive regions. Additionally, it also enables detection of multiple breakpoints occurring in cis on the same DNA molecule, thereby providing a comprehensive view of complex SVs and other CGRs and resolving their structures [65,66]. OGM also enables detection of copy-neutral events, smaller CNVs (~20 Kb), mosaic events, and SV breakpoints flanked by repetitive regions, as it still remains challenging despite improvements achieved through the integration of multiple methods (e.g., split-read, read-depth, paired-end, and assembly-based) for SV identification in short-read/long-read data [67]. Studies have also implicated the role of OGM in uncovering novel candidate genes [67] which harbor within complex SVs and are often misinterpreted by standard testing methods [5,67,68].

### 4.5. Structrual Variant Calling Using Next-Generation Sequencing Methods

The current SV callers are based upon the following approaches: the alignment-based SV callers rely on read depth, split reads, and regions with reference genome disparities [69,70]; the assembly-based callers rely on comparing different genome assemblies; and finally, the meta callers combine the output of multiple SV callers [71].

**Figure 3:**
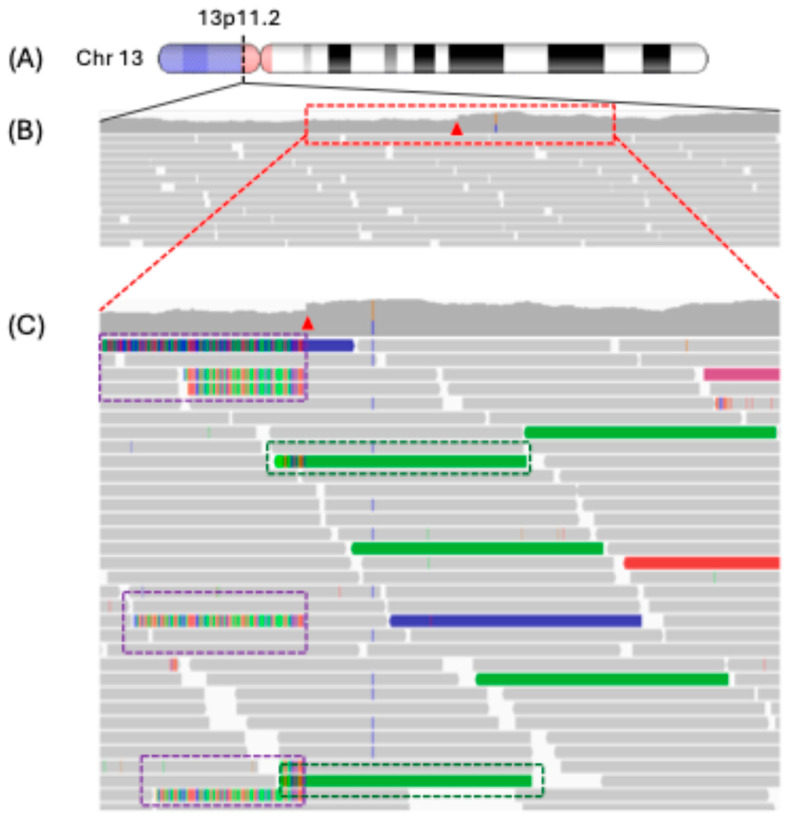
Mapping discrepancies on the Integrated Genomic Viewer (IGV). (**A**) Ideogram of chromosome 13 and the amplified view of the short arm (13p11.2) showing the locus of the proposed SV. (**B**) A magnified view of the breakpoint region on the Integrated Genomic Viewer (IGV), as outlined by a red dotted box, shows change in read depth marked by a red arrow, denoting a copy-number increase from the diploid baseline (2×) to ~3× amplification. (**C**) SV events are marked by soft-clipped reads (indicated by purple dotted boxes and shown as rainbow-colored read alignments), where individual sequencing reads align partially to separate genomic locations spanning across the breakpoint junction, thus marking the rearrangement boundary; clusters of discordant read pairs (highlighted with green dotted boxes), where paired-end reads exhibit unexpected mapping patterns (such as abnormal insert sizes or improper orientations), further support the presence and boundaries of the duplication event.

A common approach for detecting SVs in next-generation sequencing (NGS) data involves identifying mapping discrepancies during sequence alignment to the reference genome. These discrepancies include discordant reads and soft-clipped or split reads, which provide evidence for the presence of SVs (Figure 3) [72,73,74,75]. Discordant read pairs refer to paired-end reads that deviate from the expected insert size or orientation. When an SV (such as deletion, duplication, inversion, or translocation) lies between the reads, the mapping pattern is altered: the reads might align farther apart than expected (indicating a deletion) or map in the wrong orientation (indicating an inversion). This also suggests that there is a breakpoint between the reads, even though the altered sequence may not be fully captured within the reads themselves [72,76]. In contrast to this, soft-clipped and split reads offer higher resolution for mapping breakpoints. A clipped read occurs when only part of a sequencing read aligns to the reference genome, often indicating that the read spans a breakpoint. If the clipped portion can be realigned to a different genomic location, the read becomes a split read, implying that it originates from two or more distinct regions of the genome. Split reads are particularly valuable because they directly span SV breakpoints, providing nucleotide-level resolution for accurate SV characterization and the ability to generate an accurate architectural map of a given SV [74,76,77]. Some of the commonly used read depth-based SV/CNV callers include Delly [72,78], Manta [72], Sniffles [79], GRIDSS [80], LUMPY [77], Control-FREEC [81], and GROM-RD [82].

The commonly used alignment-based SV callers include PBHoney [83], NanoSV [84], NanoVar [85], Smartie-sv [74,86], Sniffles2 [87], SVIM [81,88], pbSV (pbsv—pacbio sv calling and analysis tools) [81], SKSV [89], MAMnet [90], and DeBreak [91].

Advancements in long-read sequencing and mapping technologies are making de novo genome assembly increasingly feasible for large genomes, supported by the availability of assembly-based SV callers that compare genome assemblies [92]. This unbiased approach is computationally intensive, and the called variants are often affected by repeats, polyploidy, read length, and sequencing coverage [93,94]. An assembly-based caller, novoBreak [95], leverages unmapped and partially mapped reads by clustering them and conducting local assembly around breakpoint regions, providing high precision in the results even for the somatic SV calling. *Assemblytics* [96] analyzes *MUMmer’s nucmer* [97] alignments to identify high-confidence SVs in each contig relative to a reference or another assembly. It can detect insertions and deletions ranging from 1 bp to 10 kb, with the upper limit adaptable based on the length of the unique sequence anchor and can also effectively detect tandem repeat expansions, translocations, and complex variants.

### 4.6. SV Callers from Short-Read Whole-Genome Sequencing

SV calling using short reads is based on changes in read depth, aligned clusters of discordant paired-end reads or split reads, or constructing an assembly using a combination of these [98]. For instance, DELLY utilizes split reads and discordant read pairs [85], while LUMPY further integrates read-depth data [77]. ViZCNV uses read depth and phased B-allele frequency, as well as benchmarking signals from other SV calling methods [99]. Additionally, SV callers like Manta [72], GRIDSS [80], and SvABA [100] incorporate short-read assembly to enhance detection accuracy [80], while Paragraph [101] integrates sequence graphs and SV annotations. A more comprehensive approach involves combination of the outputs of multiple SV callers and merging the results into a single call set [73,102], potentially improving SV detection [79]. Additionally, short-read SV callers cannot phase complex rearrangement breakpoints or span genomic regions with poor mapping quality. To accurately detect and phase such events, longer DNA molecules are required, utilizing linked reads, long-read sequencing, or optical genome mapping technologies [7].

### 4.7. SV Callers from Long-Read Whole-Genome Sequencing

Long-read sequencing technologies, mainly represented by Pacific Biosciences (PacBio) single-molecule real-time sequencing technology (SMRT) [103] and Oxford Nanopore Technologies (ONT) [104], enable the assembly of genomes with greater accuracy, allowing for the resolution of complex and repetitive regions that are often challenging for short-read methods and enhancing accurate detection of complex SVs located in regions with complex genomic architecture. Finally, application of long-read transcriptomics allows for the sequencing of entire transcripts, providing insights into alternative splicing events and the discovery of novel isoforms underlying genetic disorders [105]. Advancements in long-read sequencing, combined with genome-wide mapping technologies, have enabled the complete resolution and assembly of both haplotypes in the human genome [106]. It has also been noted that long-read genome analyses usually detect >20,000 SVs compared to short-read discovery approaches including 5000–10,000 SVs [1,107]. In addition to this, repeat-associated variations within SV classes, including short tandem repeats (STRs) which have been difficult to characterize using short-read sequencing technologies and are often underrepresented in the reference genome and frequently collapsed in unphased genome assemblies [106,108]. This highlights the role of long-read SV callers in improved breakpoint resolution, mapping across repetitive regions, haplotype phasing, and allele-specific detection, as well as the ability to resolve large and complex SVs [81,109,110]. Several SV detection tools have been developed for long-read sequencing data from PacBio and Oxford Nanopore Technologies (ONT). These include alignment-based SV callers, including PBSV [111], SVIM [88], Sniffles [74], CuteSV [112], SVDSS [113], SVsearcher [114,115], SVvalidation [116], and SVcnn [110].

Traditional SV callers tend to rely mostly on a single signal type. The current focus is on integrating multiple signals including read-depth, split reads, discordant paired-end reads, and assembly-based evidence for better SV calling and reducing false positives [110]. Also, the new SV callers, including CSV-Filter [117], SVcnn [116], NPSV-deep [118], SVLearn [119], and GROM [82], are written using machine learning approaches. These models can also better handle noisy or ambiguous signals and improve SV classification and breakpoint resolution [81,109,110,117,118]. Tools like PopDel [120] and GGTyper [121] were written to incorporate joint SV calling across multiple populations and samples, which can be an important component of the emerging SV callers. SV callers like WhatsHap [84], a long-read caller with haplotype-resolved SV detection and phasing, can distinguish between alleles and thus is important for understanding compound heterozygosity, imprinting, or allele-specific expression [122]. Benchmarking datasets, including the GIAB v4 SV truth sets and the T2T genome, are helping to improve and compare the SV callers [79,110,111].

### 4.8. Strand-Seq

Strand-seq is a specialized single-cell sequencing method that enables strand-specific analysis of the genome without the need for DNA amplification. This technique selectively sequences the template DNA strands, allowing generation of strand-specific libraries from individual cells [123]. By preserving the directionality of DNA strands, this technique provides valuable insights into SV detection including chromothripsis-like events, sister chromatid exchange, and genome organization, particularly in repetitive or complex regions that are challenging to resolve with conventional sequencing approaches [5,123,124,125]. In contrast to SV detection using bulk-tissue WGS, MosaiCatcher is a powerful approach for analyzing SVs at the single-cell level [125,126]. The resolution of breakpoint detection is limited to ~100 kb; Strand-seq also shows cellular heterogeneity and mosaicism, which are often obscured in bulk sequencing data, and enables the identification of haplotype-phased SVs, offering insights into the allelic context of genomic rearrangements [127].

### 4.9. High-Throughput Chromosome Conformation Capture (Hi-C)

Balanced rearrangements, such as inversions and reciprocal translocations, also contribute to clinically significant fusion genes and abnormal positioning of regulatory elements, often rendering them undetectable by standard approaches [128,129]. Hi-C has demonstrated strong potential for SV detection, as SVs can change the three-dimensional organization of the genome by bringing together regions that are normally far apart (Figure 1D) [130]. This spatial rearrangement increases the likelihood of these distant regions being ligated together, leading to an abnormal enrichment of such contacts compared to the background signal at similar genomic distances [129]. Harewood et al. used in-nucleus Hi-C, a derivative technique of the Hi-C technique, to identify known, novel as well as balanced and unbalanced chromosomal rearrangements in cell lines and human tumor samples [128]. HiC-BreakFinder [131] was one of the first tools developed to detect SVs using Hi-C, in integration with WGS and optical genome mapping, for resolving complex SVs in cancer. HiCnv [128] integrates Hi-C and WGS-based SV callers for detecting and resolving complex SVs, while HiSV [132] is a control-free method for identifying large-scale SVs from a Hi-C sample.

### 4.10. Linked-Read Sequencing

Linked-read sequencing employs microfluidics to partition high-molecular-weight (HMW) DNA molecules, typically 50 kb or larger, into individual oil emulsion droplets [133]. Within each droplet, unique droplet-specific barcodes are used to label and fragment the HMW-DNA, producing short fragments suitable for short-read sequencing. However, unlike conventional short-read sequencing, linked-read sequencing preserves long-range genomic context through these barcodes, which trace each read back to its original DNA molecule [133,134]. This long-range information is essential for allele phasing, haplotype assembly, breakpoint junction analysis, and the accurate detection of a wide range of SVs, including duplications, deletions, inversions, and translocations [135]. LinkedSV [135] was designed to detect mosaic SVs and picked up the cancer-causing SVs previously undetected by short- and long-read-sequencing-based callers. SVJAM [136], a joint SV calling method from linked-read sequencing, outperformed LongRanger, an SV calling method from long-range sequencing data. MTG-Link is a local assembly tool specifically designed for linked-read data which uses barcode information to selectively extract relevant subsets of reads, enabling targeted local assembly of specific genomic regions [137]. It supports a range of local assembly applications, including filling gaps between scaffolds, resolving clinically significant genomic regions, and reconstructing alternative sequences associated with SVs [137,138].

## 5. Challenges of Structural Variant Detection, Analysis, and Interpretation

Despite substantial evidence highlighting the importance of SVs in evolution, complex traits, and disease causation, detection of SVs is still a major challenge. An estimated 5–10% [139] of the human genome remains difficult to analyze because of highly repetitive elements which make the genomic regions highly unstable, including segmental duplications and tandem repeat centromeric arrays, as well as satellite sequences [3,12,139]. Such regions are enriched for structural complexity, giving rise to copy-number variations, inversions, complex genomic rearrangements, and gene duplications that are implicated in human evolution and disease [140,141]. These complex genomic architectural features pose significant challenges even for the current state-of-the-art SV callers designed for sequencing-based technologies, mainly in the context of uniquely mapping the reads across large homologous regions, leading to misalignment, missed variants, or a large number of false-positive calls [105,139]. SV callers also often struggle to balance false positives and false negatives, especially in low-complexity regions [73,142].

There are several sequencing and mapping errors which tend to blur the patterns of an SV [142]. SV detection typically involves analyzing changes in read depth, identifying clusters of discordantly aligned paired-end reads or split reads, constructing assemblies, or using a combination of these methods [98]. Read depth-based methods can be challenging to use, particularly when detecting balanced events (inversions or translocations), and are sensitive to GC-content bias, mapping artifacts, and sequencing coverage variability [143,144]. Discordant paired-end-read and split-read signals are effective for breakpoint detection but often generate noisy data in repetitive regions or when mapping quality is low [112]. However, assembly-based SV callers offer higher resolution and are efficient but are computationally intensive in low-complexity or poorly covered regions [143,144]. Detecting SVs with short-read sequencing is challenging because the signals indicating SVs often resemble common sequencing and alignment artifacts.

Most SV callers are optimized for simple SV calling, and thus, most of the tools might misclassify or miss the complex genomic rearrangements with multiple breakpoint junctions, and inaccurate breakpoint resolution hinders downstream functional annotation, genotyping, and interpretation [77,98]. There is still a lack of benchmarking and standardization of SV callers, as different tools can produce different SV call sets from the same data [145]. Long-read SV callers, graph-based tools, and joint-calling pipelines are computationally intensive [79].

While the ability to detect CGRs and SVs within complex genomic regions has improved significantly with advances in sequencing technologies, SV calling methods, and reference genome alignments, the bioinformatic characterization of such genomic complexities is still a major challenge. Much of the analysis, interpretation, classification, and architectural mapping of these rearrangements still relies heavily on manual curation, often requiring expertise and training to piece together the data from SV callers, raw sequence data, and/or other genomic methodologies.

## 6. Future Perspectives

A graph-based genome is a computational model that represents the genome not as a single linear sequence but as a graph where nodes represent sequences (such as genes, exons, or genomic segments) and edges represent relationships between them, such as sequence similarity or SVs [138,146]. This approach is the basis of several tools, including Variation Graph (VG) [146] and GraphAligner [147], among others, which can show multi-breakpoint events which are difficult to capture in linear genomes [146].

The reference genome agnostic approach is a method relying on more than one reference genome assembly (like GRCh38 and/or T2T-CHM13) for detecting, assembling, or interpreting genetic variants more flexibly [148,149], as many SVs are misrepresented in current references, and reads spanning across novel SVs may fail to align properly against the standard linear genome. T2T-CHM13 provides a more complete, accurate, and representative reference for variant calling using both short- and long-read sequencing across diverse human ancestries, compared to GRCh38 [149]. Reanalysis of 3202 short-read datasets from the 1000 Genomes Project revealed that T2T-CHM13 included ~182 Mbp of previously missing sequence and the removal of 1.2 Mbp of erroneously duplicated regions present in GRCh38, thereby improving variant detection by reducing both false negatives and false positives [150]. Carvalho et al. showed that several SVs (pathogenic inversions underlying neurodevelopmental phenotypes) could only be resolved using long-read whole-genome sequencing and OGM. They also emphasized the need to move beyond GRCh37 to either GRCh38, T2T-CHM13, or a reference-free assembly, since the traditional reference genome assemblies failed to interpret the variants [148,149]. Moreover, the use of a pangenome reference [151] is intended to enhance downstream analysis by minimizing the mapping biases that arise from relying on a single linear reference genome, such as GRCh38 or CHM13. This incorporates genomic diversity across multiple individuals and ancestries. The pangenome reference provides more accurate variant detection, sequence alignment, and interpretation across populations [151].

Recent advancements estimate that a healthy genome contains approximately 22,000 to 27,000 SVs which are larger than 50 bp, encompassing diverse genomic rearrangements including deletions, duplications, inversions, insertions, and complex genomic rearrangements [107,152,153,154]. The majority of these SVs go undetected by conventional cytogenetic testing or just short-read sequencing. This implies the role of combining multiple technologies such as long-read (PacBio, Oxford Nanopore), short-read (Illumina), linked-read, strand-seq, Hi-C, and OGM which can enhance SV detection compared to a short-read sequencing alone [141]. Additionally, the application of high-throughput technologies like single-cell long-read DNA sequencing enables comprehensive genomic analysis by supporting high-quality de novo assemblies, precise SV detection, haplotype phasing, resolution of complex or repetitive regions, and the profiling of epigenetic modifications [155].

Cloud-based SV callers are emerging as a useful tool in genomics due to a combination of methodologies. With the rise of population-scale sequencing [UK Biobank, All of Us, Trans-Omics for Precision Medicine (TOPMed)], cloud infrastructure offers the storage required to analyze thousands to millions of genomes in parallel [79,156]. Cloud environments make it easier to incorporate machine learning models (SV pathogenicity prediction, genotype–phenotype correlation, allele frequency) due to support from the pre-trained models. Such platforms allow the SV callers to annotate and interpret SVs in real time, which is critical for rare disease research, and multi-site consortia (AnVIL [157]). 

To understand the SVs in non-coding, inter-genic, or regulatory regions, SV callers could be built integrating gene annotations, regulatory elements, expression QTLs, and chromatin data. SV callers can also be designed to prioritize tissue-specific annotation pipelines that can link SVs to gene expression (RNA-seq), methylation, or ATAC-seq, which can reveal the functional impact of SVs and the cell-type-specific activity of SV-affected loci [158,159]. Such functional annotations can guide experimental science by prioritizing genes for CRISPR-based screens or model organisms’ studies for genotype–phenotype correlations [159]. Additionally, focusing on the development of ML-/AI-based SV impact prediction tools will aid in SV analysis. AnnotSV [160] enables comprehensive annotation of SVs by integrating functional, regulatory, and clinical data for interpreting the potential pathogenicity of SVs and filtering out likely false positives, thereby improving the reliability of downstream analyses. SVScore [161,162] is another tool which summarizes the per-base pathogenicity scores of SNPs across genomic regions affected by each SV, considering the variant type, associated gene features, and positional uncertainty.

## 7. Conclusions

SVs account for more nucleotide-level differences between two human genomes than any other type of genetic variation [140] and have been studied less extensively than SNVs, especially in low-complexity regions known as SV hotspots, which are enriched in repetitive elements, segmental duplications, and other architectural features that predispose them to structural rearrangements [111]. In this review, we highlight the evolving landscape of SV research, driven by advances in sequencing technologies as well as analysis tools. With the decreasing cost of sequencing and the advancement of SV detection methods, improvements in detection, interpretation, and characterization of SVs, enabling more comprehensive insights into the genomic architecture, disease mechanisms, and personalized medicine. Long-read sequencing, optical genome mapping, and graph-based genome representations allow us to resolve complex SVs, including regions of the genome which were poorly characterized. The continued refinement of sequencing platforms, SV calling pipelines, and interpretation methods for resolving CGRs will not only improve diagnostic yields but also deepen insights into disease mechanisms, providing new opportunities for personalized medicine and targeted therapies.

## Data Availability

Not applicable.
